# Looking Inside the Intramolecular C−H∙∙∙O Hydrogen Bond in Lactams Derived from α-Methylbenzylamine

**DOI:** 10.3390/molecules22030361

**Published:** 2017-02-28

**Authors:** Sandra Mejía, Julio M. Hernández-Pérez, Jacinto Sandoval-Lira, Fernando Sartillo-Piscil

**Affiliations:** Centro de Investigación de la Facultad de Ciencias Químicas, and Centro de Química de la Benemérita Universidad Autónoma de Puebla, 14 Sur Esq. San Claudio, San Manuel. C. P. 72570 Puebla, Mexico; mejia.cabildo@gmail.com

**Keywords:** C−H···O interaction, chiral lactams, α-methylbenzylamine, MP2

## Abstract

Recently, strong evidence that supports the presence of an intramolecular C−H···O hydrogen bond in amides derived from the chiral auxiliary α-methylbenzylamine was disclosed. Due to the high importance of this chiral auxiliary in asymmetric synthesis, the inadvertent presence of this C−H···O interaction may lead to new interpretations upon stereochemical models in which this chiral auxiliary is present. Therefore, a series of lactams containing the chiral auxiliary α-methylbenzylamine (from three to eight-membered ring) were theoretically studied at the MP2/cc-pVDZ level of theory with the purpose of studying the origin and nature of the C−Hα···O interaction. NBO analysis revealed that rehybridization at C atom of the C−Hα bond (s-character at C is ~23%) and the subsequent bond polarization are the dominant effect over the orbital interaction energy *n*_(O)_→σ*_C−Hα_ (E(2) < 2 kcal/mol), causing an important shortening of the C−Hα bond distance and an increment in the positive charge in the Hα atom.

## 1. Introduction

With the vast theoretical and experimental contributions coming from the laboratories of Desiraju [1,2,3], Steiner [4,5], and Scheiner [6,7] regarding the existence of C−H···O hydrogen bonding within a wide range of synthetic and naturally occurring compounds, this “weak” interaction has become a well-accepted axiom. Indeed, today its relevance and importance in chemistry is fundamental to the interpretation and understanding of molecular conformation (either in solution or in the solid state [8]) and of chemical transformations in solution. Although this interaction does not usually exceed 3 kcal/mol [9,10], it plays a crucial role in favoring crystallization [11,12], conformational equilibrium [13,14] or inducing selectivity [15,16,17,18,19,20,21,22]. In this context, a recent report which describes the role of the intramolecular C−Hα···O contact of chiral lactams (derived from the α-methylbenzylamine) in their spatial arrangement—both in solution and in solid state [23]—have attracted our attention, especially because this chiral auxiliary is frequently employed in asymmetric synthesis [24]. Consequently, the current theoretical study of a set of cyclic amides derived from α-methylbenzylamine attempts to further advance towards the understanding of the origin and the nature of this C−Hα···O hydrogen bonding.

## 2. Results and Discussion

**Figure 1:**
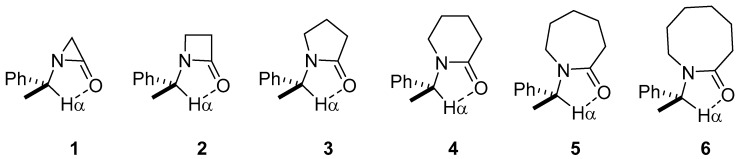
Systems studied.

If we assumed the presence of a C−Hα···O hydrogen contact, then a bicycle-type molecular structure, as shown in Figure 1, must be formed. Therefore, a set of non-substituted lactams derived from α-methylbenzylamine, going from three to eight-membered rings (**1**–**6**) were modeled.

To corroborate the presence of a C−Hα···O hydrogen bond in lactams **1**–**6**, molecular geometry analysis, NBO analysis, C−Hα stretching, and electron density analysis were used.

### 2.1. Energy and Geometrical Parameters

**Figure 2:**
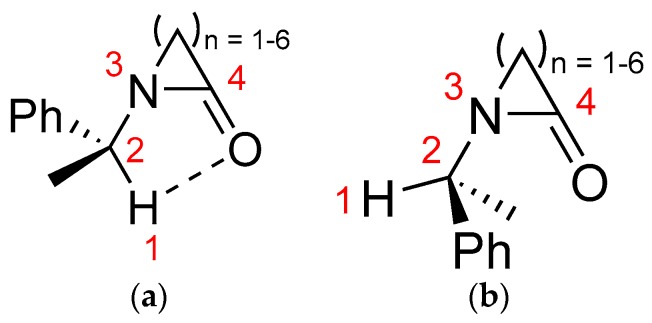
The dihedral angle Φ is formed by atoms H(1), C(2), N(3), and C(4). (**a**) Closed, when Φ presents values near 0.0°; and (**b**) open, when Φ presents values near 180°.

Similar to previous work [23], two minima were observed for lactams **1**–**6**: the closed structure **a** and the open structure **b** (Figure 2). These geometries were characterized by the dihedral angle Φ given by the H(1), C(2), N(3), and C(4) atoms. The closed geometry **a** has values Φ near to 0.0°, suggesting C-Hα···O bond formation, whereas in the open conformation **b**, the Φ angle is near to 180°, indicating the lack of Hα···O contact. Subsequently, the geometry of lactams depicted in Figure 1 were optimized in both closed and open forms, and their energy differences were determined (see Supplementary File: Tables S1–S12). The relative energies (ΔE = E_closed_ − E_open_) were corrected by zero point energies, and the results are shown in Table 1.

**Figure 3:**
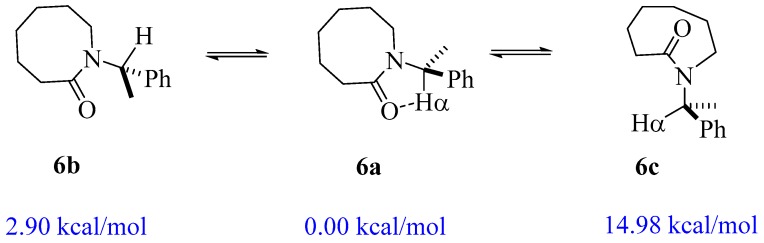
Observed conformers of lactam **6,** and their relative energies.

As expected, the aziridinone **1** does not reach the closed conformation (Φ angle of 33.415°) because of its very strained geometry. The positive value of ΔE = 1.20 kcal/mol indicates that the open geometry (Φ = 166.71°) is the preferred conformation. A similar result was observed for β-lactam **2**, although the energy difference between the closed (Φ = 28.003°) and open (Φ = 163.10°) forms is smaller (ΔE = 0.34 kcal/mol), again indicating a preferred open conformation. As the ring size increases from five to seven atoms (**3**–**5**), the closed geometries show dihedral angles closer to zero (20.410° to 8.575°) and the ΔE values are shifted from positive to negative, ranging from −2.19 to −4.04 kcal/mol. It is clear that the largest lactams prefer the closed geometry. However, the eight-membered lactam **6** has an energy difference of only −2.90 kcal/mol, even when the closed geometry has an almost planar dihedral angle Φ = 3.847°. It should be mentioned that due to the high conformational flexibility of **6**, a third minimum structure **6c** (*E*-conformer with 14.98 kcal/mol) was detected (see Figure 3). Given the geometry features and the high energy of conformer **6c**, it was decided not to include it in the current study.

The following parameters of the closed structures **1** to **6** were additionally studied: C−Hα distance, Hα···O distance, donor and acceptor atoms distance, and the C−Hα···O angle. These parameters were compared to the geometric criteria summarized by Steiner [2] for classifying hydrogen bonds.

As shown in Table 2, aziridinone **1** presents the longest Hα···O and C2···O distances (3.3600 and 3.3501 Å, respectively) with a C−Hα···O angle of 79.97° indicating lack of directionality. Along with the high energy of the closed geometry, this rules out the possibility of an intramolecular hydrogen bonding. For lactams **2** and **3**, their Hα···O bond distance (2.7605 and 2.3449 Å, respectively), C2···O distance (3.1269 and 2.8585 Å, respectively), and C−Hα···O bond angle (98.85° and 106.37°, respectively) indicate that C−Hα···O are getting closer to being identified as weak hydrogen bonds [2]. For the larger lactams **4**, **5**, and **6**, the Hα···O bond distance (from 2.1785 to 2.1390 Å) and the C2···O distance (from 2.7425 to 2.7340 Å) become even shorter, while the C−Hα···O bond angle increases from 109.07° to 111.09°. These data are in concordance with a weak C−Hα···O interaction.

An interesting trend observed in Table 2 is that as the ring size increases, the C−Hα and C2···O distances shorten. These structural changes attenuate the *n*_(O)_→σ*_C−Hα_ orbital interaction, which in turn affects the C−Hα stretching. This behavior is observed in hydrogen bonds wherein the electronegativity of the donor atom X (e.g., C2) and the H are similar. Such interactions have been called “improper” or “blue-shifting” hydrogen bonds [25,26].

### 2.2. NBO Analysis

The red and blue shifting of hydrogen bonds is attributed to a pair of correlated effects; namely, the n_(Y)_→σ*_X−H_ orbital interaction and rehybridization at the X atom with a concomitant polarization of the X−H bond [27,28,29,30]. According to Alabugin et al. [27], as the electronegativity of Z increases (Z = CH_3_, NH_2_, OH, F), so does the polarization of a C−Z bond, whereas the s-character in the carbon-centered hybrid atomic orbital of the C−Z bond is reduced. This is a direct consequence of Bent’s rule, [31] and can be observed not only in C−H bonds, but also in Si−H, S−H, and N−H bonds [32,33,34]. In the hyperconjugation, the acceptor Y donates a lone pair to the C−H bond σ* orbital, causing its lengthening and weakening, with the subsequent red-shifting of the stretching frequency. Depending on which effect dominates upon hydrogen bonding, the C−H bond will become shorter (rehybridization) or larger (hyperconjugation). To evaluate these effects in the closed lactams **2** to **6**, we used NBO analysis [35,36], and the results are displayed in Table 3. The open conformer of lactam **4** was also calculated as a reference point.

**Figure 4:**
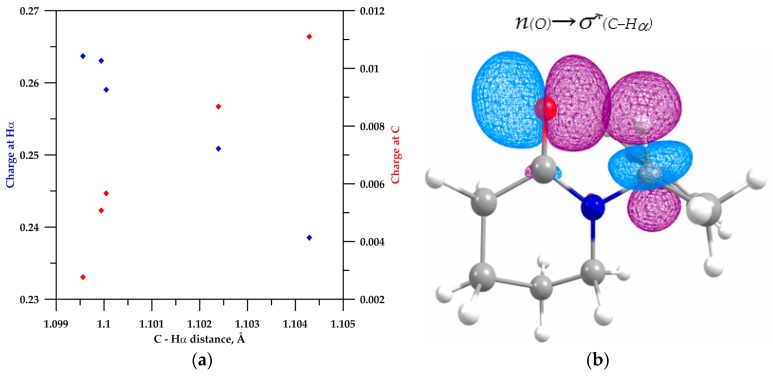
(**a**) Correlation of population of charges for H and C in the C−Hα bond with the C−Hα distance (charge of H in blue and for C in red); (**b**) NBO plots illustrate the overlap of the *n*(o) orbital and the σ*_(C−Hα)_ orbital in the conformer **4a**.

For lactams **3**–**6**, the s-character on the carbon donor and the C−Hα bond polarization increased (compared to that of open conformer **4b**). Furthermore, the C−Hα bond distance and the hydrogen and carbon charges are highly correlated; for instance, as the hydrogen charges become more positive and the carbon charges becomes less positive, the C−Hα distance shortens (see Figure 4a).

**Figure 5:**
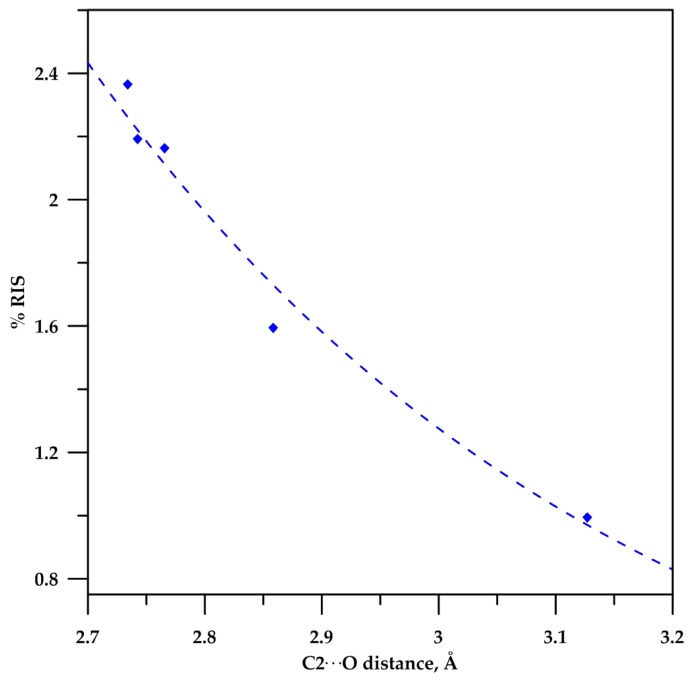
Correlation of %RIS for C−H bond with C2···O distance donor–acceptor. %RIS = 813.64Exp(−2.153*distance), R^2^ = 0.9806.

With regard to the hyperconjugation effect, the NBO calculation for β-lactam **2** did show a negligible orbital overlap *n*_(O)_→σ*_(C−Hα)_ (E(2) = 0.11 kcal/mol), which excludes the C−Hα···O hydrogen bonding. This is expected, since the geometric parameters already pointed in this direction. Cyclic amide **3** has a *n*_(O)_→σ*_(C−Hα)_ orbital interaction of 0.75 kcal/mol that suggests a weak orbital overlap, which stems from a poor C−Hα directionality (the closed conformer **3** is deviated 20.41° from the planarity). The hyperconjugative energy for lactams **4** (i.e., the energy associated to the *n*_(O)_→σ*_(C−Hα)_ interaction, see Figure 4b), **5**, and **6** are in the same order of magnitude: 1.26, 1.29, and 1.60 kcal/mol, respectively (see Table 3). These hyperconjugative energies, along with the low orbital populations σ*(C−H), are characteristic of an improper hydrogen bonding [25,26].

### 2.3. Analysis of the C−Hα Stretching

Since the blue-shifting of vibrational frequencies originates from the strong repulsion between the hydrogen atom and the acceptor, as well as the attraction between the donor and the acceptor atoms [37], it is expected to find these vibrational frequencies in the systems studied herein. Thereupon, the fundamental stretching frequencies of C−Hα bond for lactams **2**–**6** in both conformations (open and closed)—and consequently their corresponding relative infrared shift (RIS)—were calculated. The results are displayed in Table 4.

In all cases, blue-shift frequencies are observed for the closed conformers **a**, which is in accordance with the presence of the C−Hα···O interaction. According to the RIS values of 0.99% to 2.37%, the C−Hα···O bond gets stronger as the ring size increases; nevertheless, it remains within the range of weak hydrogen bonding, which is <10% [4,5,6]. It should be noted that the RIS of β-lactam **2** is only 0.99%; therefore, it can be disregarded as evidence of a C−Hα···O contact, and similarly for lactam **3**, for which showed a RIS value of 1.59%. These results are in agreement with a poor *n*_(O)_→σ*_(C−H__α__)_ orbitaloverlap.

The correlation between the donor–acceptor distance (C2···O in Table 2) and the relative infrared shift (%RIS in Table 4) for lactams **2**–**6** is more clearly recognized in Figure 5.

### 2.4. Analysis Based in Quantum Theory of Atoms in Molecules

Analysis based on topological properties of the electron density (ρ(r)) is an additional tool for the study of hydrogen bonding. In the Quantum Theory of Atoms in Molecules (QTAIM) introduced by Bader [38], the analysis of critical points provides important information about interatomic interactions. At a critical point, the gradient of the electron density vanishes, and they are classified into four groups: maxima in ρ(r) are related to nuclei, minima are associated with cage critical points, and saddle points are connected with ring or bond critical points. The bond critical points (BCP) represent the minimum in ρ(r) along the bonding direction, pointing out a chemical interaction. Koch and Popelier [39] have suggested some criteria to characterize hydrogen bonds, such as values of electron density, ρ(r) = 0.002–0.040 a.u., and its Laplacian, ∇^2^ρ(r) = 0.024–0.139 a.u.

The values of ρ(r) and its Laplacian in BCP for lactams **4** to **6** are shown in Table 5. While lactam **3** does not exhibit a BCP, larger lactams **4**, **5**, and **6** indeed show a BCP for the C−Hα···O interaction, whose values are within the stablished range [20]. Since the C−Hα···O bond is present in lactams **4**–**6**, a bicyclic structure is formed. Consequently, ring critical points inside the second cycle were found for the corresponding lactams.

In spite of lactam **3** showed some features that might be associated to the existence of a hydrogen bond, such as %RIS = 1.59 and the presence of the C−Hα bond polarization, the absence lack of BCP suggests an extremely weak hydrogen bond.

## 4. Materials and Methods

In this study, the MP2/cc-pVDZ level of theory was used. Geometries were fully optimized, and minima were confirmed through vibrational analysis. All calculations were carried out using Gaussian 09 [40]. The topological analysis of the electron density was carried out using DensToolKit program [41].

## 5. Conclusions

The theoretical study reported here states that the dominant effect that originates the C−Hα···O hydrogen bonding in cyclic amides, derived from α-methylbenzylamine, is the polarization of the C−Hα bond, which is reflected in the increment of the s-character at C atom (it increases from 22.05% to 22.87%). As the distance of the C−Hα bond shortens, the charge at the H atom increases, favoring this “improper” hydrogen bonding.

## Figures and Tables

**Table 1:** Dihedral angles and relative energies of open and close conformer.

Lactam	Φ, Open (°)	Φ, Closed (°)	ΔE (kcal/mol)
**1**	166.71	33.415	1.20
**2**	163.10	28.003	0.34
**3**	156.24	20.410	−2.19
**4**	166.56	9.837	−2.11
**5**	156.39	8.575	−4.04
**6**	157.02	3.847	−2.90

**Table 2:** Geometrical parameters of closed structures.

Lactam	O^…^H (Å)	C−H (Å)	C2^...^O (Å)	C−H^...^O (°)
**1**	3.3600	1.1105	3.3501	79.97
**2**	2.7605	1.1042	3.1269	98.85
**3**	2.3449	1.1023	2.8585	106.37
**4**	2.1785	1.0999	2.7425	109.07
**5**	2.1933	1.1000	2.7655	109.71
**6**	2.1390	1.0995	2.7340	111.09

**Table 3:** C−H Distance and NBO analysis in closed conformers. The data for **4** open (**4b**) is used as reference.

Lactam	C−Hα (Å)	s-Character % at C2	Polarization of C−Hα Bond		Charge (u.a.)		Population σ*(C−Hα)	E(2) (kcal/mol)
			% C	% H	at C	at Hα		
**4b**	1.1046	20.99	60.43	39.57	0.01615	0.20563	0.01615	-
**2**	1.1042	22.05	62.010	37.990	0.01694	0.23853	0.01694	0.11
**3**	1.1023	22.49	62.750	37.350	0.01781	0.25087	0.01781	0.75
**4**	1.0999	22.87	63.390	36.610	0.01889	0.26308	0.01889	1.26
**5**	1.1000	22.75	63.190	36.810	0.01826	0.25905	0.01826	1.29
**6**	1.0995	22.76	63.440	36.560	0.01888	0.26374	0.01888	1.60

**Table 4:** Calculated frequencies of the fundamental C−Hα stretching, and their corresponding relative infrared shifts (%RIS).

Lactam	Conformers a	Conformers b	
	ν (cm^−1^)	ν (cm^−1^)	%RIS
**2**	3105.26	3074.69	0.99
**3**	3123.34	3074.32	1.59
**4**	3145.59	3078.07	2.19
**5**	3145.03	3078.42	2.16
**6**	3147.57	3074.81	2.37

**Table 5:** Values of electron density and Laplacian of electron density in the C−Hα···O bond critical point.

Lactam	ρ (BCP)	∇^2^ρ(r) (BCP)
**4**	0.0226	0.0843
**5**	0.0219	0.0816
**6**	0.0241	0.0870

## Supplementary Materials

The following are available online. Tables S1–S12: xyz coordinates of the optimized geometry and MP2/cc-pVDZ energy of all structure study.
