# Supplementary material for: Looking Inside the Intramolecular C−H∙∙∙O Hydrogen Bond in Lactams Derived from α-Methylbenzylamine
Source: Molecules. 2017 Feb 28;22(3):361. doi: 10.3390/molecules22030361 (PMC6155423; doi:10.3390/molecules22030361)
Supplement: Supplementary file 1 [file molecules-22-00361-s001.pdf]

Supporting Information

Looking inside the intramolecular C–H···O hydrogen bond in lactams derived from α-methylbenzylamine

Sandra Mejía, Julio M. Hernández-Pérez,* Jacinto Sandoval-Lira,* and Fernando Sartillo-Piscil.*

Centro de Investigación de la Facultad de Ciencias Químicas, and Centro de Química de la Benemérita Universidad Autónoma de Puebla, México. 14 Sur Esq. San Claudio, San Manuel. C. P. 72570, Puebla, México* Correspondence: jsandovalira@gmail.com; julio.hernandez@correo.buap.mx; fernando.sartillo@correo.buap.mx; Tel.: +52 222 229500x7391.

Table S1. XYZ coordinates for conformer **1 closed (1a)** from MP2/cc-pVDZ optimization

E(MP2/cc-pVDZ) =- -516.08462473024au

E(ZPE) = 0.188714 au

Atomic	Coordinates (Angstroms)		
	X	Y	Z
7	-1.715603000	0.041147000	0.429214000
6	-2.272944000	-1.110241000	-0.068252000
6	-3.215776000	0.020050000	0.126852000
1	-3.860565000	0.146446000	1.001930000
1	-3.547979000	0.526625000	-0.791538000
6	-0.775820000	0.891726000	-0.317005000
1	-1.033522000	0.866135000	-1.396956000
8	-2.021549000	-2.213553000	-0.486380000
6	0.626856000	0.350911000	-0.144124000
6	1.517172000	0.324378000	-1.232274000
6	1.071931000	-0.079959000	1.120541000
6	2.838428000	-0.118935000	-1.061563000
6	2.389985000	-0.528542000	1.290618000
6	3.277752000	-0.545971000	0.201539000
1	1.171959000	0.647283000	-2.220662000
1	0.373199000	-0.080038000	1.962246000
1	3.520895000	-0.139145000	-1.916551000
1	2.724886000	-0.869439000	2.275026000

1	4.304758000	-0.898362000	0.335376000
6	-0.903474000	2.325456000	0.194846000
1	-1.934672000	2.695387000	0.069195000
1	-0.640028000	2.364897000	1.263429000
1	-0.221976000	2.987376000	-0.362011000

Table S2. XYZ coordinates for conformer **2 closed (2a)** from MP2/cc-pVDZ optimization

E(MP2/cc-pVDZ) = - 555.31599843500au

E(ZPE) = 0.219948 au

Atomic	Coordinates (Angstroms)		
	X	Y	Z
7	-1.524891000	0.395539000	0.023221000
6	-2.259041000	-0.761044000	-0.191442000
6	-2.660524000	-0.824156000	1.298246000
6	-1.672818000	0.360718000	1.488660000
1	-3.717786000	-0.596826000	1.496086000
1	-2.376434000	-1.763711000	1.793757000
1	-0.740510000	0.086588000	2.011977000
1	-2.091176000	1.283557000	1.920732000
8	-2.431950000	-1.435715000	-1.187902000
6	-0.468703000	0.967413000	-0.802926000
1	-0.709963000	0.625728000	-1.824936000
6	0.867461000	0.355178000	-0.406235000
6	1.093504000	-1.000672000	-0.726310000
6	1.853376000	1.057356000	0.310672000
6	2.282912000	-1.637022000	-0.344778000

6	3.046638000	0.420164000	0.694231000
6	3.264399000	-0.926993000	0.368771000
1	0.323610000	-1.551778000	-1.277988000
1	1.704758000	2.109974000	0.566944000
1	2.446531000	-2.686776000	-0.606972000
1	3.806378000	0.979994000	1.248320000
1	4.194545000	-1.420739000	0.665435000
6	-0.525754000	2.491985000	-0.757794000
1	0.263233000	2.932224000	-1.387091000
1	-1.505700000	2.834666000	-1.122635000
1	-0.396344000	2.866476000	0.270467000

Table S3. XYZ coordinates for conformer **3 closed (3a)** from MP2/cc-pVDZ optimization

E(MP2/cc-pVDZ) = - 594.53169371368au

E(ZPE) = 0.250500 au

Atomic	Coordinates (Angstroms)		
	X	Y	Z
6	-2.740702000	0.017425000	1.634593000
6	-2.836465000	-1.149968000	0.647992000
6	-2.010715000	-0.686510000	-0.549877000
7	-1.288826000	0.419718000	-0.139941000
6	-1.372398000	0.632357000	1.300429000
1	-2.819804000	-0.280155000	2.690439000
1	-3.855823000	-1.425129000	0.342868000
1	-2.345723000	-2.056935000	1.044697000
8	-1.957443000	-1.202055000	-1.661203000

1	-1.315455000	1.703446000	1.550437000
1	-0.548653000	0.111762000	1.828177000
1	-3.530109000	0.755575000	1.419484000
6	-0.170624000	0.911515000	-0.946184000
1	-0.360041000	0.490704000	-1.947334000
6	-0.193284000	2.435071000	-1.040772000
1	-1.147366000	2.761685000	-1.481861000
1	-0.092964000	2.915058000	-0.054594000
1	0.632164000	2.790606000	-1.677151000
6	1.127343000	0.313577000	-0.425158000
6	2.041141000	1.033548000	0.366397000
6	1.389193000	-1.045482000	-0.700995000
6	3.197438000	0.411196000	0.868234000
6	2.541612000	-1.667955000	-0.200223000
6	3.450097000	-0.940309000	0.588008000
1	1.864366000	2.089624000	0.589443000
1	0.677103000	-1.606376000	-1.315981000
1	3.901324000	0.985326000	1.478709000
1	2.734746000	-2.720500000	-0.429447000
1	4.351746000	-1.423065000	0.976663000

Table S4. XYZ coordinates for conformer **4 closed (4a)** from MP2/cc-pVDZ optimization

E(MP2/cc-pVDZ) = - 633.71481268323au

E(ZPE) = 0.280124 au

Atomic	Coordinates (Angstroms)		
	X	Y	Z

7	-1.015400000	0.419663000	-0.242846000
6	-1.814225000	-0.481821000	-0.912024000
6	-3.020889000	-1.042235000	-0.166915000
6	-3.373341000	-0.331083000	1.136498000
6	-2.085648000	-0.121371000	1.927037000
6	-1.179451000	0.825145000	1.152310000
1	-3.853048000	-1.048480000	-0.888145000
1	-2.781095000	-2.101881000	0.034564000
1	-4.110596000	-0.918782000	1.707515000
1	-3.834849000	0.650911000	0.925255000
1	-2.277987000	0.307020000	2.924468000
1	-1.576022000	-1.090974000	2.071331000
1	-0.174720000	0.860664000	1.610241000
1	-1.600248000	1.848048000	1.200257000
8	-1.571689000	-0.855433000	-2.062567000
6	0.162417000	0.953580000	-0.948234000
1	0.035138000	0.613655000	-1.986564000
6	0.171891000	2.480747000	-0.932706000
1	-0.761545000	2.860174000	-1.377025000
1	0.261119000	2.894197000	0.084002000
1	1.019310000	2.857549000	-1.526531000
6	1.413913000	0.291557000	-0.392831000
6	2.393447000	0.976770000	0.349094000
6	1.574319000	-1.093222000	-0.614928000
6	3.511386000	0.293641000	0.860951000
6	2.688508000	-1.774967000	-0.107167000
6	3.660587000	-1.082879000	0.637023000
1	2.299873000	2.052208000	0.524642000

1	0.818374000	-1.622381000	-1.205249000
1	4.265412000	0.840847000	1.435209000
1	2.802987000	-2.846982000	-0.295479000
1	4.531724000	-1.613154000	1.033319000

Table S5. XYZ coordinates for conformer **5 closed (5a)** from MP2/cc-pVDZ optimization

E(MP2/cc-pVDZ) = -672.89420166620au

E(ZPE) = 0.309329 au

Atomic	Coordinates (Angstroms)		
	X	Y	Z
7	0.986824000	0.904704000	-0.213809000
6	1.970795000	0.594515000	0.707198000
6	3.158645000	-0.176570000	0.143392000
6	0.963434000	0.262593000	-1.525764000
6	2.828629000	-1.663597000	-0.165303000
6	0.645600000	-1.244148000	-1.479295000
6	1.330927000	-1.962247000	-0.312991000
1	3.949240000	-0.108211000	0.903503000
1	3.364973000	-1.980813000	-1.077495000
1	1.933486000	0.427132000	-2.022400000
1	0.966390000	-1.687853000	-2.440183000
1	3.527310000	0.336158000	-0.759246000
1	0.214498000	0.783272000	-2.142474000
1	3.218473000	-2.286472000	0.656927000
1	-0.444184000	-1.392897000	-1.398085000
1	1.186953000	-3.050656000	-0.427342000

1	0.820040000	-1.685775000	0.625716000
6	-0.222783000	1.597206000	0.260951000
1	0.013225000	1.862010000	1.302248000
6	-0.460979000	2.887778000	-0.520933000
1	-1.355097000	3.404239000	-0.136681000
1	0.408617000	3.552508000	-0.404210000
1	-0.609586000	2.711405000	-1.598069000
6	-1.391386000	0.628271000	0.280148000
6	-1.446403000	-0.322923000	1.320867000
6	-2.374695000	0.590298000	-0.725872000
6	-2.448891000	-1.303073000	1.344376000
6	-3.380887000	-0.391314000	-0.705184000
6	-3.416119000	-1.344620000	0.324636000
1	-0.688990000	-0.281610000	2.111824000
1	-2.365564000	1.329073000	-1.532778000
1	-2.479397000	-2.032727000	2.159574000
1	-4.138674000	-0.409561000	-1.494724000
1	-4.199429000	-2.108427000	0.339767000
8	1.904328000	0.921658000	1.894179000

Table S6. XYZ coordinates for conformer **6 closed (6a)** from MP2/cc-pVDZ optimization

E(MP2/cc-pVDZ) = -712.07715276754au

E(ZPE) = 0.338532 au

Atomic	Coordinates (Angstroms)		
	X	Y	Z
7	-0.706818000	1.090155000	0.285748000

6	-1.763033000	1.014034000	-0.598032000
6	-1.613251000	-1.958704000	1.229912000
6	-3.049354000	0.381833000	-0.099351000
6	-1.939758000	-1.922814000	-0.277331000
6	-3.148394000	-1.051969000	-0.641661000
1	-3.137245000	0.385612000	0.995646000
1	-2.525958000	-1.748221000	1.820399000
1	-2.140973000	-2.947239000	-0.635487000
1	-1.314150000	-2.985166000	1.504664000
1	-3.872893000	0.988693000	-0.506402000
1	-1.049030000	-1.577350000	-0.832559000
1	-4.070568000	-1.519574000	-0.251830000
1	-3.246458000	-1.000972000	-1.739413000
6	-0.693452000	0.481047000	1.617505000
1	-1.610788000	0.754211000	2.165338000
1	0.132472000	0.961181000	2.165776000
6	-0.468887000	-1.038501000	1.682025000
1	0.440580000	-1.286805000	1.109045000
1	-0.242787000	-1.268179000	2.740821000
6	0.552351000	1.674715000	-0.216112000
1	0.303574000	1.994216000	-1.238395000
6	0.955551000	2.914778000	0.580175000
1	0.149771000	3.663007000	0.526285000
1	1.151621000	2.702797000	1.642980000
1	1.871159000	3.352606000	0.151362000
6	1.610975000	0.592617000	-0.315080000
6	2.638465000	0.429211000	0.631302000
6	1.502537000	-0.338113000	-1.370136000

6	3.527950000	-0.655978000	0.539204000
6	2.387603000	-1.421153000	-1.464803000
6	3.400168000	-1.587483000	-0.502810000
1	2.753601000	1.147251000	1.448745000
1	0.708614000	-0.200241000	-2.113093000
1	4.321473000	-0.772925000	1.283911000
1	2.291400000	-2.134338000	-2.289454000
1	4.092010000	-2.432308000	-0.572371000
8	-1.681815000	1.401443000	-1.768131000

Table S7. XYZ coordinates for conformer **1 open (1b)** from MP2/cc-pVDZ optimization

E(MP2/cc-pVDZ) =- -516.08719731075au

E(ZPE) = 0.188714 au

Atomic	Coordinates (Angstroms)		
	X	Y	Z
7	1.841156000	0.432227000	0.603121000
6	2.114578000	-0.878833000	0.348047000
6	1.606305000	-0.590073000	1.713529000
1	2.239536000	-0.451397000	2.595557000
1	0.566743000	-0.896797000	1.901050000
6	0.815588000	1.269191000	-0.051134000
1	0.720064000	2.178558000	0.567459000
8	2.419159000	-1.661404000	-0.524278000
6	-0.532422000	0.576863000	-0.084852000
6	-1.572527000	1.022588000	0.752460000
6	-0.758597000	-0.541880000	-0.915815000

6	-2.820635000	0.378457000	0.756363000
6	-2.002885000	-1.190157000	-0.907840000
6	-3.037704000	-0.731250000	-0.075041000
1	-1.402119000	1.887043000	1.403734000
1	0.040174000	-0.917031000	-1.561685000
1	-3.620033000	0.741460000	1.409424000
1	-2.165346000	-2.056355000	-1.556351000
1	-4.007676000	-1.237242000	-0.074556000
6	1.345752000	1.649656000	-1.432983000
1	2.301307000	2.187271000	-1.334620000
1	1.521814000	0.749232000	-2.041528000
1	0.619455000	2.293536000	-1.952509000

Table S8. XYZ coordinates for conformer **2 open (2b)** from MP2/cc-pVDZ optimization

E(MP2/cc-pVDZ) = -555.31653187919 au

E(ZPE) = 0.219934au

Atomic	Coordinates (Angstroms)		
	X	Y	Z
7	1.511595000	0.336345000	0.480718000
6	2.256666000	-0.442988000	-0.395633000
6	2.766611000	-1.322114000	0.766396000
6	1.770867000	-0.523102000	1.647238000
1	2.545497000	-2.391821000	0.642185000
1	3.829730000	-1.180253000	1.007331000
1	2.195542000	0.015612000	2.509649000
1	0.886223000	-1.103139000	1.962132000

8	2.389667000	-0.431629000	-1.605204000
6	0.398940000	1.253056000	0.268151000
1	0.327218000	1.871783000	1.181838000
6	-0.906145000	0.491842000	0.109619000
6	-1.995016000	0.739792000	0.964012000
6	-1.038445000	-0.476043000	-0.908137000
6	-3.204110000	0.041622000	0.805853000
6	-2.243876000	-1.175990000	-1.064796000
6	-3.330061000	-0.918445000	-0.210051000
1	-1.895885000	1.487497000	1.759137000
1	-0.193556000	-0.680234000	-1.574080000
1	-4.043565000	0.244151000	1.478022000
1	-2.337171000	-1.924418000	-1.857739000
1	-4.269204000	-1.465610000	-0.335117000
6	0.687684000	2.170443000	-0.924001000
1	-0.146518000	2.877669000	-1.050854000
1	1.617287000	2.735955000	-0.752965000
1	0.807206000	1.582977000	-1.844836000

Table S9. XYZ coordinates for conformer **3 open (3b)** from MP2/cc-pVDZ optimization

E(MP2/cc-pVDZ) = -594.52808242270 au

E(ZPE) = 0.250377 au

Atomic	Coordinates (Angstroms)		
	X	Y	Z
6	-2.591584000	-1.811214000	0.276891000
6	-3.137873000	-0.490230000	0.825098000

6	-2.002712000	0.497491000	0.550430000
7	-1.278954000	-0.022939000	-0.498122000
6	-1.708113000	-1.358491000	-0.896959000
1	-3.365918000	-2.529431000	-0.030277000
1	-3.387926000	-0.499238000	1.894826000
1	-4.028663000	-0.152275000	0.266153000
8	-1.790033000	1.550726000	1.145559000
1	-0.832738000	-2.013477000	-1.045126000
1	-2.273219000	-1.320352000	-1.849209000
1	-1.956912000	-2.290656000	1.040067000
6	-0.113975000	0.583454000	-1.148242000
1	-0.085750000	0.137515000	-2.159392000
6	-0.240639000	2.100255000	-1.321272000
1	-1.175103000	2.346693000	-1.850763000
1	-0.246623000	2.614805000	-0.352918000
1	0.611998000	2.456577000	-1.920820000
6	1.165459000	0.182741000	-0.435591000
6	1.362299000	0.533386000	0.916729000
6	2.169490000	-0.534356000	-1.110878000
6	2.546479000	0.168282000	1.573654000
6	3.357343000	-0.897588000	-0.453657000
6	3.547286000	-0.546163000	0.891558000
1	0.580088000	1.086540000	1.445798000
1	2.021652000	-0.807688000	-2.162065000
1	2.691059000	0.444270000	2.622810000
1	4.130157000	-1.456009000	-0.990817000
1	4.470082000	-0.827908000	1.407544000

Table S10. XYZ coordinates for conformer **4 open (4b)** from MP2/cc-pVDZ optimization

E(MP2/cc-pVDZ) = -633.71131100369 au

E(ZPE) = 0.279991 au

Atomic	Coordinates (Angstroms)		
	X	Y	Z
7	1.092274000	0.158589000	0.617627000
6	1.608126000	0.860161000	-0.455983000
6	2.893556000	0.321449000	-1.078153000
6	3.034716000	-1.197228000	-1.027976000
6	2.795936000	-1.652066000	0.407041000
6	1.387608000	-1.264153000	0.834485000
1	2.923748000	0.718016000	-2.103626000
1	3.730225000	0.789680000	-0.527443000
1	4.029309000	-1.505737000	-1.389637000
1	2.286267000	-1.668055000	-1.691404000
1	2.909396000	-2.743500000	0.515874000
1	3.531593000	-1.170911000	1.075944000
1	1.249343000	-1.468585000	1.909968000
1	0.647566000	-1.886731000	0.291564000
8	1.125206000	1.916455000	-0.867096000
6	-0.173078000	0.591413000	1.255635000
1	-0.191697000	0.018092000	2.199681000
6	-0.203025000	2.069630000	1.653302000
1	0.724796000	2.331758000	2.186960000
1	-0.304123000	2.733079000	0.788080000
1	-1.055021000	2.225118000	2.334885000
6	-1.386712000	0.145034000	0.457229000

6	-1.759040000	0.782801000	-0.745887000
6	-2.158521000	-0.940854000	0.915525000
6	-2.877040000	0.334820000	-1.465911000
6	-3.279478000	-1.387455000	0.196406000
6	-3.641278000	-0.748565000	-0.999427000
1	-1.158893000	1.615545000	-1.118702000
1	-1.882623000	-1.436061000	1.853826000
1	-3.155644000	0.837788000	-2.397270000
1	-3.868531000	-2.230378000	0.570917000
1	-4.513907000	-1.090799000	-1.563945000

Table S11. XYZ coordinates for conformer **5 open (5b)** from MP2/cc-pVDZ optimization

E(MP2/cc-pVDZ) = -672.88746672667 au

E(ZPE) = 0.338432 au

Atomic	Coordinates (Angstroms)		
	X	Y	Z
7	0.744192000	-0.134635000	0.005159000
6	1.273397000	1.133319000	-0.060193000
6	2.467576000	1.297225000	-1.001715000
6	1.265931000	-1.198393000	-0.847381000
6	3.713134000	0.415337000	-0.696821000
6	2.534817000	-1.828191000	-0.256983000
6	3.485873000	-0.750794000	0.276420000
1	2.728520000	2.362859000	-0.938665000
1	4.126297000	0.021152000	-1.642682000
1	1.477364000	-0.796269000	-1.849862000

1	3.030859000	-2.428198000	-1.041557000
1	2.114324000	1.123332000	-2.032525000
1	0.468577000	-1.946708000	-0.983807000
1	4.496175000	1.062176000	-0.267785000
1	2.269786000	-2.522946000	0.559874000
1	4.459441000	-1.210432000	0.518986000
1	3.088786000	-0.351154000	1.225663000
6	-0.338784000	-0.512005000	0.935641000
1	-0.225441000	-1.604527000	1.052833000
6	-0.209262000	0.086843000	2.340373000
1	-0.943662000	-0.411911000	2.993524000
1	0.800252000	-0.097075000	2.743412000
1	-0.392810000	1.167031000	2.344261000
6	-1.704056000	-0.271486000	0.317954000
6	-2.564788000	-1.355249000	0.062309000
6	-2.132277000	1.033960000	-0.003841000
6	-3.833721000	-1.148995000	-0.504775000
6	-3.397572000	1.239265000	-0.573384000
6	-4.252354000	0.151531000	-0.824574000
1	-2.240282000	-2.371393000	0.315446000
1	-1.462782000	1.876846000	0.188459000
1	-4.491631000	-2.002162000	-0.696999000
1	-3.720628000	2.255385000	-0.820571000
1	-5.239064000	0.317847000	-1.267281000
8	0.824887000	2.093048000	0.573122000

Table S12. XYZ coordinates for conformer **6 open (6b)** from MP2/cc-pVDZ optimization

E(MP2/cc-pVDZ) = -712.07242607746au

E(ZPE) = 0.309041 au

Atomic	Coordinates (Angstroms)		
	X	Y	Z
7	-0.527376000	0.080702000	-0.024896000
6	-1.014116000	-1.179736000	-0.283747000
6	-3.412871000	1.475595000	-0.296677000
6	-2.254189000	-1.266545000	-1.159256000
6	-3.600541000	0.179898000	0.520886000
6	-3.519723000	-1.118061000	-0.296056000
1	-2.265899000	-0.527645000	-1.972508000
1	-3.695512000	1.298056000	-1.352066000
1	-4.591524000	0.198316000	1.006843000
1	-4.118839000	2.238798000	0.074013000
1	-2.239047000	-2.266908000	-1.617058000
1	-2.861750000	0.154123000	1.340864000
1	-4.401108000	-1.180993000	-0.959862000
1	-3.574106000	-1.980774000	0.390617000
6	-0.870343000	1.260772000	-0.822763000
1	-1.099587000	0.968392000	-1.857976000
1	0.045398000	1.874227000	-0.885635000
6	-2.009520000	2.099279000	-0.237493000
1	-1.767084000	2.337511000	0.815376000
1	-2.026623000	3.063753000	-0.778364000
6	0.532421000	0.324428000	0.978380000
1	0.399052000	1.386303000	1.252902000
6	0.380696000	-0.471468000	2.278616000
1	-0.646201000	-0.376953000	2.668352000

1	0.602251000	-1.535254000	2.140182000
1	1.076251000	-0.045705000	3.020129000
6	1.915765000	0.198164000	0.366610000
6	2.377938000	-1.038401000	-0.132271000
6	2.760947000	1.321750000	0.295511000
6	3.660356000	-1.136616000	-0.691934000
6	4.046887000	1.222350000	-0.262216000
6	4.498854000	-0.009849000	-0.758314000
1	1.721280000	-1.911126000	-0.082483000
1	2.410584000	2.283552000	0.688290000
1	4.009779000	-2.099881000	-1.076636000
1	4.691776000	2.105265000	-0.309745000
1	5.498888000	-0.093082000	-1.194515000
8	-0.553963000	-2.203030000	0.233487000
